# Dysregulation of Metabolic Peptides Precedes Hyperinsulinemia and Inflammation Following Exposure to Rotenone in Rats

**DOI:** 10.3390/cells14020124

**Published:** 2025-01-16

**Authors:** Vandana Zaman, Denise Matzelle, Naren L. Banik, Azizul Haque

**Affiliations:** 1 Ralph H. Johnson Veterans Administration Medical Center, 109 Bee Street, Charleston, SC 29401, USA; zamanv@musc.edu (V.Z.); baniknl@musc.edu (N.L.B.); 2 Department of Neurosurgery, Medical University of South Carolina, 96 Jonathan Lucas Street, Charleston, SC 29425, USA; matzeldd@musc.edu; 3 Department of Pharmacology and Immunology, Medical University of South Carolina, 173 Ashley Avenue, Charleston, SC 29425, USA

**Keywords:** rotenone, hormonal peptides, inflammation, hyperinsulinemia, obesity, diabetes

## Abstract

Rotenone, a naturally occurring compound derived from the roots of tropical plants, is used as a broad-spectrum insecticide, piscicide, and pesticide. It is a classical, high-affinity mitochondrial complex I inhibitor that causes not only oxidative stress, α-synuclein phosphorylation, DJ-1 (Parkinson’s disease protein 7) modifications, and inhibition of the ubiquitin-proteasome system but it is also widely considered an environmental contributor to Parkinson’s disease (PD). While prodromal symptoms, such as loss of smell, constipation, sleep disorder, anxiety/depression, and the loss of dopaminergic neurons in the substantia nigra of rotenone-treated animals, have been reported, alterations of metabolic hormones and hyperinsulinemia remain largely unknown and need to be investigated. Whether rotenone and its effect on metabolic peptides could be utilized as a biomarker for its toxic metabolic effects, which can cause long-term detrimental effects and ultimately lead to obesity, hyperinsulinemia, inflammation, and possibly gut–brain axis dysfunction, remains unclear. Here, we show that rotenone disrupts metabolic homeostasis, altering hormonal peptides and promoting infiltration of inflammatory T cells. Specifically, our results indicate a significant decrease in glucagon-like peptide-1 (GLP-1), C-peptide, and amylin. Interestingly, levels of several hormonal peptides related to hyperinsulinemia, such as insulin, leptin, pancreatic peptide (PP), peptide YY (PYY), and gastric inhibitory polypeptide (GIP), were significantly upregulated. Administration of rotenone to rats also increased body weight and activated macrophages and inflammatory T cells. These data strongly suggest that rotenone disrupts metabolic homeostasis, leading to obesity and hyperinsulinemia. The potential implications of these findings are vast, given that monitoring these markers in the blood could not only provide a crucial tool for assessing the extent of exposure and its relevance to obesity and inflammation but could also open new avenues for future research and potential therapeutic strategies.

## 1. Introduction

Rotenone, a naturally occurring compound derived from the roots of tropical plants, is used as a broad-spectrum insecticide, piscicide, and pesticide. Studies have shown that farming communities and war veterans exposed to pesticides have a high incidence of developing neurological dysfunctions [1,2,3,4]. Rotenone toxicity affects various targets and molecular pathways in the body. The mode of action of rotenone is via specific mitochondrial complex I inhibition, causing cellular respiration inhibition and reactive oxygen species production [5]. Rotenone is highly lipophilic, easily crosses cellular membranes and the blood–brain barrier, and targets the central nervous system (CNS) [5,6,7]. In animal models, rotenone exposure has been linked to changes in gut microbiota, alterations in serum amino acids, inhibition of microtubule assembly, loss of enteric neurons, increased GFAP+ enteric glial cells, and α-synuclein pathology [8,9,10,11]. Moreover, Heinze and collaborators reported histopathological changes and altered liver metabolic processes caused by rotenone treatment in rats [12]. The effect of rotenone on the gut is evident by its action on gastrointestinal (GI) motility [13]. Studies suggest GI motility is a complex dynamic process, and highly coordinated interactions occur between the smooth muscle layers, enteric nervous system (ENS), CNS, gastric hormones, peptides, and the microbiome [14,15,16]. In addition, GI motility is an essential biological requirement following food intake for maintaining nutrients and hormonal output in the blood, which maintains nutrients and stable blood sugar for biological activities. Following food intake, the release of gastric inhibitory and excitatory hormones from the intestine and pancreas regulates biological processes. While rotenone can alter liver metabolic processes in rats [12], the effects of rotenone on the hormonal peptides that regulate the gut–brain axis and inflammation remain largely unknown.

The gut–brain axis is a bidirectional hormonal and neural signaling pathway connecting the gut and the brain [17]. It contributes to metabolic homeostasis, which maintains a stable metabolic state in the host. Many metabolic hormones or peptides act on the gut–brain axis and regulate immediate satiety signals, long-term food intake, energy metabolism, and body weight [16]. Hormonal peptides are short chains of amino acids that can also play important roles in metabolism and various biological processes, modulating inflammation and diseases. Metabolic hormones may become chronically dysregulated in diseases linked to obesity and could impact the progression of inflammatory diseases such as diabetes, cardiovascular, and neurodegenerative diseases. For example, hormones such as leptin upregulate multiple inflammatory cytokines and growth factors [18], mediating the production of other proinflammatory factors that need to be regulated. Additionally, in obese patients, elevated levels of circulating leptin make individuals more susceptible to developing type 2 diabetes, cardiovascular diseases, or degenerative diseases [19]. Maintaining glucose homeostasis is a dynamic physiological process. Insulin release is a complex phenomenon, and ongoing research in this field is crucial to further our understanding of the mechanism and factors involved in the process. Some known regulatory hormones involved in insulin release are leptin, incretins, estrogen, melatonin, and growth hormones [20,21,22,23,24,25,26]. Thus, this study aims to investigate whether rotenone, a natural insecticide on crops and livestock, dysregulates metabolic peptides and inflammatory factors in vivo in rats. The incretin hormones, glucagon-like peptide-1 (GLP-1) and glucose-dependent insulinotropic peptide (GIP), also inhibit GI motility and gastric emptying. Importantly, they work together to reduce postprandial hyperglycemia by glucose-dependent insulin secretion and inhibiting glucagon release [27], suggesting a potential therapeutic role in managing metabolic disorders.

This study collected plasma samples from control and rotenone-treated rats to measure important metabolic hormonal peptides that regulate energy homeostasis and metabolism. Blood samples were considered essential in investigating rotenone-induced adverse effects in rats as a biomarker for identifying metabolic hormonal disorders. Our results suggest remarkable changes in amylin, insulin, leptin, C-peptide, PP, PYY, and incretin hormones GLP-1 and GIP. Along with investigating the changes in this metabolic panel, spleen samples were also examined to monitor the presence of peripheral inflammatory markers such as CD68, CD4, and TNF-α in both control and rotenone-treated rats. Additionally, some peripheral pro-inflammatory and anti-inflammatory factors were also measured in the plasma to assess pathogenic status in the rats following administration of rotenone. The administration of rotenone also influences insulin resistance and obesity, suggesting inflammation, neuronal dysfunction, and degeneration could occur in the long run. Detecting changes in these metabolic hormonal protein markers may indicate an ongoing disease process. Due to physiological and metabolic differences between humans and rats, further in-depth studies are needed to determine if these metabolic markers could serve as potential diagnostic markers for ongoing diseases. Furthermore, our findings underscore the critical role of blood parameters in evaluating the extent of exposure to environmental toxins and their potential future implications for individual health and well-being.

## 2. Materials and Methods

### 2.1. Animals

Three- to four-month-old male Lewis rats (300–350 gm body weight, ENVIGO, Indianapolis, IN, USA) were housed in the animal facility under standard conditions (12 h light–dark cycles, 23 °C, and 55% relative humidity). Rats had ad libitum access to food and water. The animals were handled and cared for in compliance with the guidelines of the National Institutes of Health (NIH, Bethesda, MD, USA) *Guide for the Care and Use of Laboratory Animals* (NIH publication 80–23, revised 1996) and approved (ACORP 643) by the Institutional Animal Care and Use Committee (IACUC) of the Ralph H. Johnson Veteran Medical Center of South Carolina, Charleston, SC, USA.

### 2.2. Rotenone Treatment

The rats were split into two treatment groups: (1) control (vehicle) and (2) rotenone. Rotenone (Sigma-Aldrich, St. Louis, MO, USA) was administered subcutaneously (s.c.) at a dose of 2 mg/kg body weight daily for four days and then every other day for six days. A concentrated solution (50×) of rotenone was prepared using dimethylsulfoxide (DMSO) from Sigma-Aldrich (St. Louis, MO, USA), and sunflower oil (Authentic Menu, Toronto, ON, Canada) was used to prepare the working emulsified dilution. The dosage was prepared freshly every other day and stored in amber-colored glass vials. The body weights of rats were measured before and after the treatments (one month post-treatment). The data were collected from repeated experiments (at least 2 times), and each group consisted of 4–9 rats.

### 2.3. Analysis of Metabolic Peptides

One month after the last injection, rats were sacrificed according to the approved protocol. Deeply anesthetized (ketamine + xylazine; Patterson Veterinary, Ocala, FL, USA) rats were decapitated, and blood was collected in EDTA-coated glass tubes (BD Vacutainer™, Thermo Fisher Scientific, Waltham, MA, USA). Plasma was separated, and aliquots were stored at −80 °C for each group at the end of the experiment [28,29]. Plasma samples were analyzed for metabolic hormonal peptides by Rat Metabolic Hormone 10-Plex Discovery Assay (Eve Technologies, Calgary, NW, Canada).

### 2.4. Tissue Processing

Organs were dissected and fixed in 4% paraformaldehyde or stored in dry ice before transferring to −80 °C. Fresh frozen spleens were processed to make cryo-blocks in Tissue-Tek O.C.T. compound (optimal cutting temperature, Sakura Finetek, St. Torrance, CA, USA), and 20 µm coronal cryosections were cut and sampled on glass slides.

### 2.5. Immunostaining

Cryosections were processed for immunostaining as described [30,31]. The sections were washed in phosphate-buffered saline (0.01 M PBS, pH 7.4) +0.01% Triton-x 100 (PBST) and incubated in 8% normal horse serum (GIBCO, Thermo Fisher Scientific, Waltham, MA, USA) for 30 min at room temperature to block non-specific binding. Following this, blocking sections were incubated in primary antibodies overnight at 4 °C. The following primary antibodies were used as a marker for inflammation: anti-CD68 (ab283654, Abcam, Waltham, MA, USA), anti-CD4 (sc-19641, Santa Cruz Biotechnology, Dallas, TX, USA), and anti-TNF-α (PA1-40281, Invitrogen, Thermo Fisher Scientific, Waltham, MA, USA) antibodies. The next day, sections were washed with PBS and then incubated in Texas Red (TI-2000, Vector Laboratories, Newark, CA, USA) or VectaFluor Duet Immunofluorescence Double Labeling Kit (DK-8828, Vector Laboratories, Newark, CA, USA) for an hour. Slides were mounted with VECTASHIELD Antifade Mounting Medium with DAPI (H-1200). Images were evaluated and captured with an Olympus IX73 microscope, followed by analysis using ImageJ software (1.54f, NIH). The ‘Cell Counter’ plugin in ImageJ (Fiji) was used to count the CD4- and TNF-α-stained cells. However, to count the CD68-expressing cells in the spleen sections, ‘Analyze particles’ was used after converting the image into a binary image.

### 2.6. Statistical Analysis

Microsoft Excel and GraphPad Prism (version 6.0) software were used for statistical analyses. Data were expressed as mean ± STDEV and analyzed by a Student’s two-tailed paired *t*-test and one-way ANOVA with the Bonferroni test for the statistical difference between the groups. A *p*-value < 0.05 was determined to be statistically significant for all calculations.

## 3. Results

### 3.1. Dysregulation of Metabolic Peptides After Administration of Rotenone in Rats

**Figure 1:**
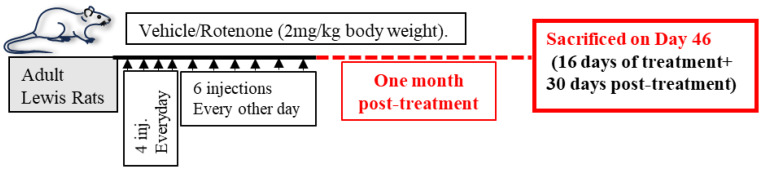
Schematic representation of the experimental design. Young adult Lewis rats received vehicle or rotenone (2 mg/kg body weight) in respective treatment groups. Rotenone treatment groups received 10 sub-cutaneous injections of rotenone (total 20 mg/kg body weight). The change in body weight was measured before the treatment (C-pre-treatment, and R-pre-treatment) and one month post-treatment (C-post-treatment and R-post-treatment). Rats were sacrificed one month post-treatment, and samples were collected.

As described in the Materials and Methods and presented in the schematic diagram (Figure 1), Lewis rats were treated with vehicle (control group) or rotenone (treatment group).

Plasma samples from these two groups were analyzed for a panel of metabolic hormonal peptides by Rat Metabolic Hormone 10-Plex Discovery Assay. The number of samples tested were 6 to 10 in each group. Due to the removal of outliers and the out-of-range readings during multiplex detection, the number of samples changed as shown in the figures. Data obtained suggest significant changes in hormonal peptides in the rotenone treatment group compared to the control group. Most of the parameters strongly indicate hyperinsulinemia in the experimental group with the rotenone treatment as described below.

### 3.2. Changes in Incretins in Rat Plasma by Rotenone

**Figure 2:**
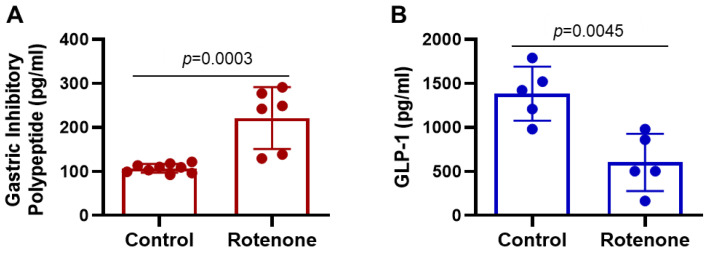
Alterations of hormonal peptides GIP and GLP-1 in rats following rotenone administration. Plasma samples from control and rotenone-treated rats were analyzed by Metabolic Hormone 10-Plex Discovery Assay. (**A**) GIP level shows a highly significant increase (*p* = 0.0003) in the plasma of rotenone-treated rats compared to the control rats. (**B**) Analysis of GLP-1 showed a significant reduction of this metabolic peptide in the plasma of rotenone-treated rats (*p* = 0.0045) compared to the control group. N = 5–8.

Alterations GLP-1 and GIP in rats after rotenone treatment were observed (Figure 2). These gut-derived hormones, called incretins, are members of the glucagon superfamily and are released by endocrine cells located in the epithelium of the small intestine in response to nutrient ingestion [23,32]. GLP-1 is synthesized by enteroendocrine L-cells [32], whereas the GIP is synthesized by enteroendocrine K cells [33]. These peptides stimulate beta cells of the pancreas to increase insulin secretion in a glucose-dependent manner [22]. The administration of rotenone significantly reduced the plasma level of GLP-1 (*p* < 0.005) compared to control animals (Figure 2A). Contrary to GLP-1, the GIP levels were significantly increased (*p* < 0.01) in the rotenone treatment group compared to the control rats (Figure 2B). These data suggest that rotenone may dysregulate incretins, altering metabolic processes in the host.

### 3.3. Significant Increase in Anorectic Hormones in Rat Plasma by Rotenone

**Figure 3:**
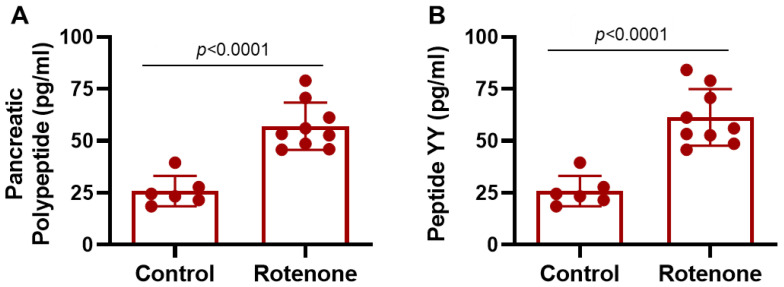
Rotenone administration elevated anorectic gut hormones, pancreatic polypeptide and peptide YY, in rats. Both pancreatic polypeptide (**A**) and peptide YY (**B**) hormonal peptides were significantly increased (*p* < 0.0001) in the plasma of the rotenone-treated group compared to the control group. N = 6–9.

PP and PYY are structurally closely related to anorectic gut hormones and constitute the PP fold peptide family [34,35]. PP, a 36-amino-acid peptide hormone secreted by the F cells, is also called PP cells of the islets of Langerhans of the pancreas [36,37,38]. The PP secretion is increased locally within islets after eating. There, it suppresses somatostatin secretion and lifts the brake on insulin secretion. The critical functions of this hormone include regulating various digestive processes, such as inhibition of pancreatic secretion, gallbladder contraction, gastric motility, and acid secretion [39]. A highly significant increase in PP level in the plasma was detected in the rotenone-treated rats (*p* < 0.0001) compared to the control group (Figure 3A). The gut-derived hormone PYY is known to be released from L cells primarily in the terminal ileum, colon, rectum, and proximal ileum to a lesser extent following food intake [35,40,41]. The functions of this peptide are to reduce gastric secretion and delay gastric emptying, and it is supposed to be a satiety signal. Most importantly, PYY contributes to the ‘ileal-brake’ phenomenon by inhibiting gastric and upper small-bowel motility to increase the absorption of nutrients [42]. Analysis of rat plasma indicates a highly significant increase in PYY (Figure 3B) in the rotenone-treated rats compared to control rats (*p* < 0.0001).

### 3.4. Alterations of Insulin, C-Peptide, Amylin, and Leptin by Administration of Rotenone

**Figure 4:**
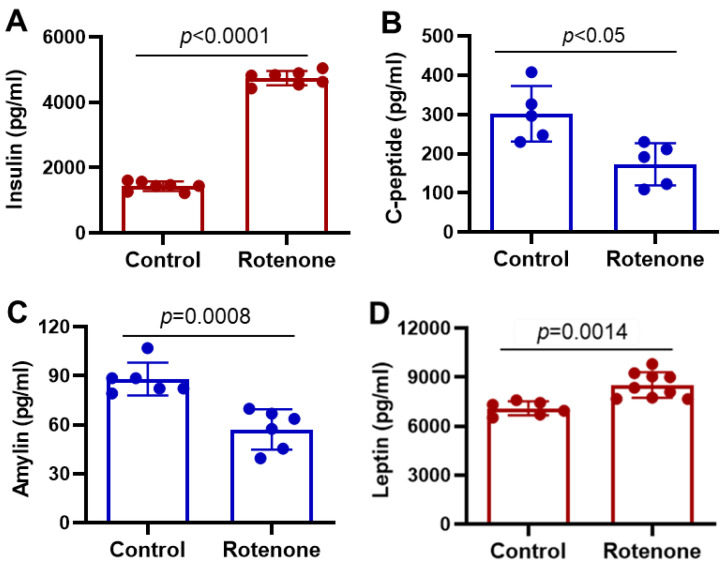
Effects of rotenone on incretin levels in rat plasma one month post-treatment. (**A**) Metabolic profile in the plasma demonstrates a highly significant increase (*p* < 0.0001) in the insulin level following rotenone treatment compared to the control group. (**B**) Meanwhile, the C-peptide level in rotenone-treated rats was significantly decreased (*p* < 0.05). (**C**) Amylin levels were also decreased significantly (*p* < 0.0008) in the rotenone group compared to the control group. (**D**) However, the plasma level of leptin hormone was increased significantly (*p* < 0.001) in rotenone-treated rats compared to the control group. These data suggest that rotenone disrupts incretin levels, promoting inflammation and insulinemia. N = 6–9.

Insulin, a peptide hormone, is secreted by β-cells of islets of Langerhans in the pancreas and regulates glucose metabolism [20]. The presence of glucose after food intake primarily induces insulin secretion. Contrary to the GLP-1 level in the plasma, the insulin levels were significantly increased (Figure 4A, *p* < 0.0001) following rotenone treatment in rats compared to the rats in the control group.

C-peptide (connecting peptide), a 31-amino-acid polypeptide, is secreted along with insulin by β-cells of islets of Langerhans in the pancreas [24]. The low level of C-peptide is a characteristic feature of type 1 diabetes [43]. A critical functional role of C-peptide is the inhibition of high glucose-induced reactive oxygen species (ROS) production and apoptosis of endothelial cells through the inhibition of transglutaminase and the AMP-activated protein kinase α (AMPKα) pathway [44,45]. The detection of C-peptide indicates elevated insulin levels in the plasma because it is co-secreted with insulin. Our data suggest that the C-peptide level in the plasma is not as high as insulin in the rotenone-treated rats as compared to the control rats (Figure 4B). However, it was significantly reduced (*p* < 0.05) in the rats treated with rotenone.

Amylin, a 37-amino-acid peptide, is co-secreted with insulin by β-cells in the pancreas after elevated blood glucose [46]. The intestine, stomach, lung, and hypothalamus also secrete it [20,46]. The amylin level in the plasma was significantly low (*p* < 0.001) in the rats treated with rotenone compared to controls (Figure 4C).

Leptin is a multifunctional 16 kDa peptide hormone secreted by adipocytes. The critical functions of leptin are appetite control, energy reserve metabolism, immune response, and reproductive processes [47]. Leptin is a pleiotropic gut hormone, but it can also be considered a pro-inflammatory cytokine that belongs to the family of long-chain helical cytokines and may regulate insulin synthesis and secretion from pancreatic β-cells [48]. Due to its dual nature as a hormone and cytokine, the most crucial function of leptin appears to be linking the neuroendocrine and the immune system [18]. Leptin also reduces hepatic glucose production and decreases glucagon levels [49]. Thus, its regulation is important in maintaining homeostasis. Unlike amylin, leptin levels were increased significantly (*p* < 0.001) in rotenone-treated rats compared to the control group (Figure 4D).

### 3.5. Rotenone Treatment Increased Body Weight in Rats

**Figure 5:**
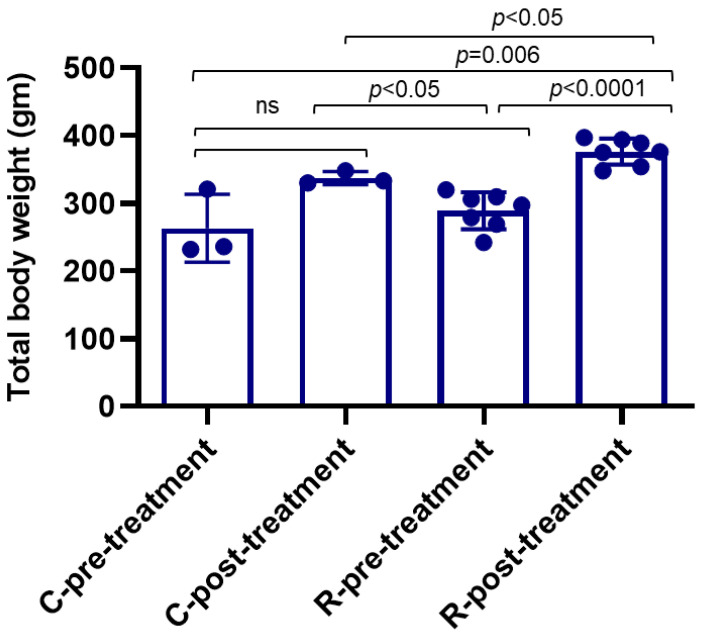
Rotenone treatment increased rat body weight. The change in body weight was measured before the treatment (C-pre-treatment and R-pre-treatment) and one month post-treatment (C-post-treatment and R-post-treatment). The data showed that the rats in the rotenone post-treatment (R-post-treatment) group gained substantial body weight, and it was significantly increased (*p* < 0.0001) compared to before starting the rotenone treatment (R-pre-treatment) and to the post-vehicle control (C-post-treatment) group (*p* < 0.005). Body weights of the control pre-treatment and the rotenone pre-treatment group are not significantly different (*p* < 0.05) at the beginning of the experiment. Moreover, no significant difference (ns) in body weights was detected in the control groups, C-pre-treatment and C-post-treatment. N = 3–7.

Dysregulation of hormonal peptides may disrupt body weight. Thus, the body weight of each rat was measured before the administration of rotenone, and animals were distributed in both groups randomly. Then, the body weight was taken one month post-rotenone treatment and analyzed. These data indicated a significant increase (*p* < 0.0001) in the body weight of rats one month post-rotenone treatment compared to body weight at the beginning of the treatments (Figure 5). The control rats treated with the vehicle did not show significant differences in body weight when compared before and after the vehicle treatment (*p* > 0.05). The rats post-rotenone treatment also showed a significant increase in body weight compared to post-vehicle treatment (control) rats (*p* < 0.005). Meanwhile, the control and rotenone rats showed no significant difference (*p* = 0.47) in body weight before administering rotenone, suggesting increased obesity in rotenone-treated rats.

### 3.6. Rotenone Treatment Activated Macrophages and Inflammatory CD4^+^ T Cells in Rats

**Figure 6:**
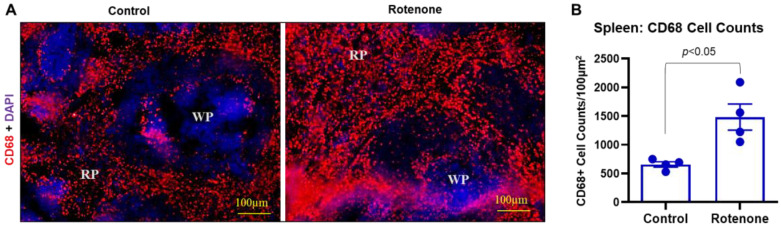
Administration of rotenone promoted activation of macrophages in rats. (**A**) Immunostaining of CD68 in cryosections of spleens from control and rotenone treatment groups. There was a distinct upregulation of CD68-positive cells in the rat spleen’s red pulp (RP) area following rotenone treatment compared to the vehicle treatment control group. (**B**) Counting of CD68-stained cells by ImageJ software showed a significant increase (*p* < 0.05) in CD68-positive cells in the rotenone treatment group compared to the control. N = 4.

The spleen comprises two functionally and morphologically distinct compartments, the red pulp (RP) and the white pulp (WP), shown in Figure 5. The critical function of the red pulp is to filter blood, removing foreign material and damaged and exhausted erythrocytes [50]. In rodents, it is a site of hematopoiesis, particularly in fetal and neonatal animals, underscoring the spleen’s role in the body’s development. Most importantly, the spleen, the largest secondary lymphoid organ, contains about one-fourth of the body’s lymphocytes and initiates immune responses to blood-borne antigens [51,52]. CD68 is a 110 kD glycosylated type I membrane protein expressed by macrophages and is widely used as a pan-macrophage marker. Red pulp macrophages distinctly express CD68. Figure 5 shows CD68-positive cells conspicuously distributed in the spleen and localized mainly in the RP area. The spleen from the rats with rotenone treatment stained with CD68 shows noticeable hyperplasia in the RP (Figure 6A).

**Figure 7:**
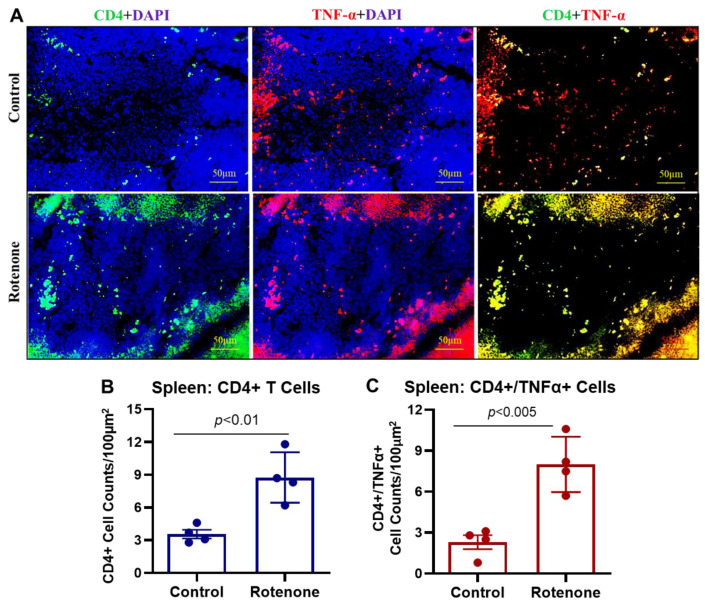
Rotenone treatment promoted activation of inflammatory CD4^+^ T cells in rats. (**A**) Immunostaining for the presence of CD4 and TNF-α in spleens from control and rotenone treatment groups counterstained with DAPI. Expansion of CD4^+^ T cells in the spleen following rotenone treatment was noticeable compared to control. These CD4^+^ T cells also expressed TNF-α, indicating inflammatory changes in the rotenone-treated animals. (**B**,**C**) Counting of cells expressing CD4 (**B**) and CD4/TNF-α markers (**C**). The rotenone treatment group showed a significant increase (*p* < 0.01) in the CD4^+^ T cell population in the spleen compared to the spleens from the control group. Moreover, these CD4^+^ T cells also co-expressed increased TNF-α in rotenone-treated rats compared to the control group (*p* < 0.005). N = 4.

The cell count data by ImageJ software confirmed an increase in CD68-positive cells in the red pulp of the spleen from rotenone-treated rats, and this increase is significantly different (*p* < 0.05) as compared to the control rats (Figure 6B). Additional immunostaining data show the markers of inflammation, such as co-expression of CD4 and TNF-α in the cryosections of the spleen (Figure 7A). While CD4 and TNF-α markers were detected in the control spleen, the rotenone-treated rats showed a significant increase in TNF-α and CD4 expression levels (*p* < 0.01, Figure 7B). Quantitative analysis of immunohistochemistry data showed a significant increase in the presence of both CD4 and TNF-α markers (co-localized, yellow) (*p* < 0.005) in the rotenone-treated rats as compared to the control group (Figure 7C), suggesting generation of inflammatory CD4^+^ T cells in the rats by administration of rotenone. Additional analysis by multiplex assays showed several other pro-inflammatory and anti-inflammatory factors in the rat plasma samples are also influenced by rotenone treatment (Table 1). The alteration of these factors indicates rotenone administration may promote the production of pro-inflammatory factors while attenuating anti-inflammatory factors in rats.

One month after treatment with rotenone, changes in several pro-inflammatory and anti-inflammatory factors in plasma were compared to the vehicle-treated control group. Quantitative analysis of these factors was determined by multiplex arrays. Factors with significant differences (*p*-value < 0.05) were presented.

## 4. Discussion

Changes in plasma metabolic panels could be an excellent clinical marker to track exposure to environmental toxicants and their long-lasting impact on the biological system. Our study suggests that the disruption of metabolic homeostasis following rotenone treatment is reflected in the changes in metabolic markers in rats’ plasma. Analysis of rat plasma samples revealed hyperinsulinemia and obesity in the rotenone-administered rats. Among different metabolic hormones, incretins GLP-1 and GIP are gut-derived peptides released following ingestion of nutrients. GLP-1 suppresses appetite and food intake in both animals and humans [25,53,54]. Our data showed low levels of GLP-1 in the rotenone-treated rats which correlated with increased body weight, an important finding in this study. We also examined the incretin hormone GIP, a 42-amino-acid enzyme that enhances insulin secretion by activating pancreatic islets following food intake, especially after glucose, fat, and amino acids [25]. GIP promotes pancreatic β-cell growth, differentiation, and survival [55]. The finding of increased GIP and insulin in the plasma of rotenone-treated rats suggests that the elevated level of GIP might have been responsible for the upregulation of insulin called hyperinsulinemia. These novel findings from our current study bring an exciting dimension to understanding the metabolic effects of rotenone in an animal model. One of the critical functions of GLP-1 is its anti-inflammatory properties in type 2 diabetes mellitus (T2DM) [56]. The significant reduction of GLP-1 levels due to rotenone treatment may lead to increased peripheral inflammation. Since the GLP-1 receptors (GLP-1Rs) are located on neurons in the brains of rodents and humans, and additionally on microglia and astrocytes in mice [55,57,58], comprehensive action of rotenone on different pathways related to GLP-1 in the CNS cannot be ruled out in rotenone-treated rats.

GLP-1-based therapies are now well-known choices for the treatment of obesity and type 2 diabetes [59,60]. Another gut-derived hormone we assessed in our study is PP, which is supposed to act as a food intake regulator. The circulating PP level is low in obese children [61]. The vagal nerve is a major stimulator of PP secretion, which can be blocked with atropine [14,62]; this suggests that the gut–brain axis plays a significant role in regulating peripheral metabolic activities. The elevated level of PP may be detected in pancreatic endocrine tumors and people with vascular risk factors, such as type 2 diabetes [37]. A significant increase in the PP hormone levels in rotenone-treated rats suggests that this increased PP might have played a role in slow gastric motility as suggested in the literature [8]. However, the rotenone-treated rats with higher levels of PP had increased body weight compared to the control. This unexpected finding suggests that this hormone might not be essential in regulating body weight homeostasis in rats, which needs to be investigated further. Like PP, the PYY levels in plasma were also significantly higher in rotenone-treated rats. Since GLP-1 inhibits the release of PYY [63], the increased level of PYY may have been caused by the reduced level of GLP-1 in these rats. Given that the rotenone treatment in the rats increased the level of the PYY in the plasma, these rats should not have gained body weight. However, these rats gained substantial body weight, indicating another key regulatory hormone is playing a detrimental role in a pathway regulating body weight. While data obtained in rats could differ from those in humans, reports suggest that PYY levels may not change in human subjects who are fasting or post-prandial [40,41,64]. Since PYY reduces food intake by modulating appetite circuits in the hypothalamus [62], the significant increase in the level of this peptide in the rotenone-treated overweight rats indicates possible gut–brain axis dysfunction.

Since C-peptide is co-secreted with insulin by β-cells of the pancreas, the low level of C-peptide in contrast to an increased insulin level is puzzling in the rats treated with rotenone in our present study. The discrepancy in plasma levels of insulin and C-peptide in these rats indicates possibly compromised insulin clearance in the liver due to rotenone toxicity. Another possible reason for the dysregulation of insulin and C-peptide could be the low levels of GLP-1 in rotenone-treated rats because GLP-1 stimulates glucose-induced insulin secretion [23,65]. The pancreatic β-cells express receptors for GLP-1 and D2-like dopamine [66,67,68], suggesting their involvement in insulin secretion. Dopamine also emerges as a key player in regulating β-cells [66,69], and the rotenone-induced loss of midbrain dopaminergic neurons is well-reported [9,31,70,71,72,73]. Thus, our study indicates that rotenone might have targeted the dopaminergic machinery in the β-cells of the pancreas and caused an increase in insulin levels in the rats. These findings also underscore the pivotal role of GLP-1 in maintaining homeostasis in the peripheral and central nervous systems, indicating the critical bidirectional role of the gut–brain axis in health and diseases.

Amylin level in the plasma is co-related with diabetes [74]. It is deficient in type 1 diabetes and relatively higher in insulin-requiring type 2 diabetes [46]. A significant reduction of amylin following rotenone treatment is detected in rats’ plasma. Contrary to amylin, insulin, co-secreted with amylin, was significantly high, indicating that insulin clearance dysfunction most probably occurs in rotenone-treated rats. The low amylin levels detected in our study following rotenone treatment might cause increased body weight. Another metabolic hormone known for its crucial role in the gut–brain axis is leptin. The circulating level of leptin concentration directly correlates with total stored body fat, and its levels are decreased during starvation [75]. In our current study, rotenone-treated rats had significantly elevated leptin and insulin levels. Circulating leptin crosses the blood–brain barrier via a receptor-mediated transport process and acts on satiety center neurons in the hypothalamus [76]. The elevated leptin level in the plasma following rotenone treatment and increased body weight in these rats suggest that cellular signaling dysregulation due to this peptide hormone may have played an important role in weight gain.

Insulin resistance is one of the leading health complications in treating patients with type 2 diabetes and obesity and is also linked to hypertension, hyperlipidemia, and atherosclerosis [77,78,79,80]. Glucose homeostasis is maintained not only by insulin secretion but also by insulin sensitivity and clearance. In this study, the detection of hyperinsulinemia in rotenone-treated rats suggests a possible metabolic disruption in the insulin regulation pathway due to rotenone’s effect on the peripheral dopaminergic system. In this challenging situation, the body’s intricate system of insulin secretion may increase, or insulin clearance may compromise as a compensatory response to maintain glucose levels [77,81]. This adaptive response underscores the body’s resilience during metabolic challenges. However, this prolonged dysregulated hyperinsulinemia may become detrimental to other regulatory pathways, ultimately leading to the onset of diabetes. In our study, rats treated with rotenone initially showed reduced gastric emptying and reduced food consumption, which were recovered in 5–7 days. The analysis of body weight revealed a significant gain in body weight in rotenone-treated animals one month post-treatment compared to the pre-rotenone treatment body weight and the control group (post-treatment). Altered levels of metabolic hormones might have contributed to this increase in body weight in rotenone-treated rats.

Immune cells may have a robust peripheral inflammatory response towards rotenone. The increased number of CD68-positive macrophages in the spleen of the rats in the rotenone group compared to the control rats suggests ongoing inflammatory changes in the peripheral immune system following rotenone exposure. Moreover, these peripheral inflammatory changes were also supported by the detection of the increased presence of TNF-α-positive CD4^+^ T cells in the spleen of the rats with rotenone treatment. The increased levels of pro-inflammatory cytokine TNF-α found here in rotenone-treated rats secreted by Th1 cells could be related to inflammation and insulin resistance [82]. Table 1 shows significant changes in the levels of pro-inflammatory factors in the plasma. These alterations support our findings of the presence of inflammatory markers in the periphery following rotenone treatment in rats. Studies have shown that diabetes is a metabolic disorder with an inflammatory response [83,84]. The systemic and local inflammatory responses are important in developing and progressing diabetes, in which CD4^+^ T cells and macrophages could be closely involved. Future investigation will analyze these subsets of CD4^+^ T cells and their functional role in the peripheral systems of metabolic disorders by rotenone.

**Figure 8:**
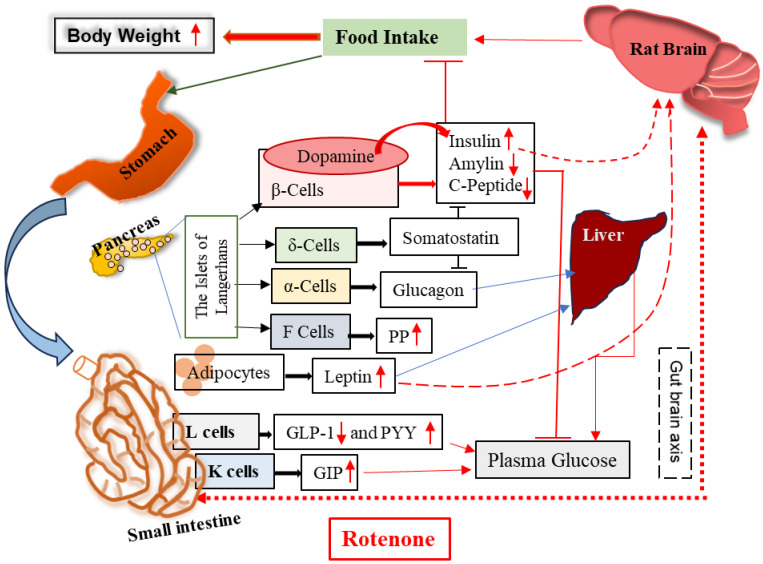
Administration of rotenone disrupts metabolic hormones and influences gut–brain axis and hyperinsulinemia. The observed low levels of GLP-1 and amylin indicate a potential dysregulation of appetite and blood sugar levels. This dysregulation may be associated with increased body weight and hyperinsulinemia. Furthermore, the hyperinsulinemia observed in the rotenone-treated rats indicates that the peripheral toxicity caused by rotenone may have disrupted regulation of insulin secretion mediated by dopamine. Red arrows indicate levels and pathways affected by rotenone treatment. Black arrows indicate the cell types and the metabolic hormones they secret.

The interaction of the host and environmental factors is a complicated phenomenon. Various intrinsic and extrinsic factors, such as diet, age, sex, gut microbiota, and infection-induced inflammation, may play critical roles in determining the host response to environmental toxicants [17,85,86,87,88,89]. Older adults are at a higher risk of cardiovascular diseases linked to type 2 diabetes and insulin resistance compared to younger adults [86]. Additionally, gut–brain axis stimulation by diet also influences the regulation of metabolic peptide secretion [87]. Figure 8 illustrates the changes in the metabolic pathways of rats following treatment with rotenone. As data evaluated in the present study were acquired from young adult rats, the age and sex of the host may influence the outcome of the findings. Additionally, rats and humans have physiological and metabolic differences; therefore, one must be cautious in interpreting the findings from the rotenone study. Overall, the metabolic profile of rotenone-treated rats indicates hyperinsulinemia and possible development of obesity. These plasma level differences in hormonal peptides after rotenone administration may provide clinical clues of disturbed signaling and inflammation at the cellular level in the periphery and in the CNS. The novelty of these findings in our study, particularly the implications for obesity, diabetes, and neurological disorders, brings an exciting dimension to understanding the metabolic effects of rotenone treatment and opens up new avenues for further research.

## Figures and Tables

**Table 1:** Analysis of select pro-inflammatory and anti-inflammatory factors in rat plasma following administration of rotenone.

Pro-Inflammatory/Anti-Inflammatory Factors	Control (pg/mL)	Rotenone (pg/mL)	*p*-Value
RANTES	2048.0 ± 273.8	5807.7 ± 2661.0	*p* = 0.00019
LIX	2555.5 ± 589	4125.2 ± 738.7	*p* = 0.0000058
IL-1β	48.6 ± 16.8	56.7 ± 30.6	*p* = 0.451
GF-CSF	55.72 ± 20.17	61.3 ± 24.0	*p* = 0.673
VEGF	11.9 ± 1.5	19.19 ± 8.18	*p* = 0.0135
IL-4	68.22 ± 16.6	33.01 ± 19.0	*p* = 0.00004
IL-10	119.01 ± 26.6	84.3 ± 12.15	*p* = 0.0325
IL-13	43.9 ± 14.6	22.17 ± 14.8	*p* = 0.0011
EGF	613.9 ± 55.4	97.0 ± 27.0	*p* = 0.0002

## Data Availability

The data used to support the findings of this manuscript are available from the corresponding authors upon reasonable written request after the publication.
